# Survey on Medical Students’ Attitudes Toward Medical Practice Just Before Clinical Clerkship in Japan

**DOI:** 10.7759/cureus.52899

**Published:** 2024-01-25

**Authors:** Nobuyasu Komasawa, Masanao Yokohira

**Affiliations:** 1 Community Medicine Education Promotion Office, Faculty of Medicine, Kagawa University, Takamatsu, JPN; 2 Department of Medical Education, Faculty of Medicine, Kagawa University, Takamatsu, JPN

**Keywords:** medical student, objective structured clinical examination, confidence, clinical clerkship, medical practice

## Abstract

Introduction: The present study examined the confidence in essential medical practices during clinical clerkship (CC) and performance in preparing education for medical students who are just starting CC at our university.

Methods: We conducted a survey using questionnaires with 105 fourth-year medical students just before starting CC. This questionnaire analysis consists of the confidence in objective structured clinical examination (OSCE) and computer-based testing (CBT) performance toward essential medical practice recommended in the "Report on Medical Practice during Clinical Clerkship" by the Japanese Ministry of Health, Labor, and Welfare and medical safety for CC.

Results: The response rate was 67.6% (71/105). As for the performance in each OSCE theme, the confidence in basic clinical technique was significantly smaller compared to chest or abdominal examination, medical interview, and emergency response (p<0.05 each). Medical interviews showed stronger confidence compared to gynecological, breast, or rectal examinations and prostate palpitation among medical examinations (p<0.05 each). In the basic technique during CC, skin disinfection showed stronger confidence compared to other techniques (p<0.05 each). On surgical technique during CC, surgical hand washing and gown technique significantly showed stronger confidence compared to skin suture, suture removal, bleeding control, and surgical assistant (p<0.05 each).

Conclusion: Our results suggest that medical students just before CC have less confidence in invasive medical practice during CC. As medical practice by student doctors becomes public, further systematic basic skill training both before and during CC is warranted.

## Introduction

Ensuring a seamless progression in medical education, where undergraduate medical students gradually acquire entrustable professional activities in medical practice until they become postgraduates, is imperative within the framework of outcome-based education [1,2]. To accomplish this, medical educators need to establish an effective clinical training curriculum for undergraduate medical students, enabling them to transition smoothly into their basic clinical skill training as postgraduates [3].

The training for clinical skills is structured as a clinical clerkship (CC) [4], differing from the traditional observation-based clinical training. CC students actively engage as members of the medical team, participating in real medical practice and care under the supervision of doctors or other medical professionals [5]. Given the opportunity to perform a specific range of medical procedures under the guidance and monitoring of teaching doctors [6], students are expected to acquire practical clinical skills. They are required to master basic physical examination and accurate medical chart documentation skills, as well as the ability to present at conferences. The curriculum for clinical training in diagnoses and treatment for CC students is determined by each hospital department [7].

Starting in 2023, student doctors will attain public certification for medical treatment in Japan [8]. Due to this change, the objective structured clinical examination (OSCE) and computer-based testing (CBT) before CC are anticipated to have more rigorous evaluations, as both assessments ensure the basic minimum clinical competency.

In the context of CC, where student doctors actively participate in medical practice, it's crucial to consider not only medical laws but also factors such as invasiveness or potential embarrassment to patients [9]. The Japanese Ministry of Health, Labor, and Welfare has categorized the medical practices that students should experience [10]. They divided medical practices into essential and recommendation items in the "Report on Medical Practice during Clinical Clerkship" guidelines [11].

While various studies have assessed the performance of medical students during CC [12], the focus has primarily been on specific skills. Previous studies have explored the correlation between the accomplishment of OSCE or CBT and performance during CC [13]. However, there has been no investigation into the confidence levels in OSCE and CBT just before CC or the confidence regarding various medical practices for coming CC. Therefore, we have undertaken a survey to examine the confidence levels of medical students just before CC regarding essential medical practice in the Japanese medical context.

In this study, we assessed the confidence levels related to essential medical practices among medical students who are about to commence their CC. Additionally, we also evaluated the confidence in accomplishment on OSCE and CBT, which are prerequisites for starting CC.

## Materials and methods

Ethical considerations

This research received approval from the Research Ethics Committee of the Faculty of Medicine, Kagawa University (approval no. 2023-071). The survey was administered to fourth-year medical students at our university who had successfully completed OSCE or CBT. This took place on December 14, 2023, coinciding with the orientation lectures for the upcoming CC. Prior to the survey, verbal informed consent was obtained from the students by a medical instructor, and this process was witnessed by medical clerks. All fourth-year medical students were briefed about the study's nature and objectives, with a guarantee of anonymity. Additionally, students were informed of their option to withdraw from the study by notifying the investigator within a week after completing the survey. It was emphasized that withdrawing from the study would not impact their academic progress in any way. Notably, all fourth-year medical students in Japan are over 21 years old; hence, the study did not involve any minors [14].

Inclusion and exclusion criteria and study measures

We included all 105 fourth-year medical students at our university and excluded none of them. We conducted a survey using questionnaires to gauge the attitudes of medical students toward their confidence in accomplishing OSCE and CBT, as well as their attitudes toward diverse medical practices and the training environment during the CC. The questionnaire's structure is outlined in Table 1 and comprises three main sections: Theme 1, confidence in the OSCE or CBT they just completed; Theme 2, confidence regarding various medical practices they are about to begin during CC; and Theme 3, confidence in the medical safety associated with CC.

**Table 1:** Questionnaire contents to medical students just before starting clinical clerkship (CC) Image Credit: Nobuyasu Komasawa

Serial no.	Theme	Content
1	OSCE and CBT	Confidence of total OSCE or CBT accomplishment, confidence on each OSCE theme (8 themes)
2	Medical practice	(a) Medical examination (11 items), (b) general technique (10 items), (c) surgical technique (8 items), (d) laboratory examination (10 items), (e) emergency response (5 items), (f) treatment (5 items)
3	Medical safety	Medical safety (patient safety, infection control, information management)

The questions were developed based on essential medical practice outlined in the "Report on Medical Practice During Clinical Clerkship" guidelines provided by the Japanese Ministry of Health, Labor, and Welfare [11]. Medical students rated their confidence using a visual analog scale (VAS), ranging from 0 mm (indicating an extreme lack of confidence) to 100 mm (indicating an extremely high level of confidence) [15]. The questionnaire's content was evaluated by three professionals in medical education. Subsequently, a pilot test involving four medical clerks from our department was conducted.

Study population

**Figure 1:**
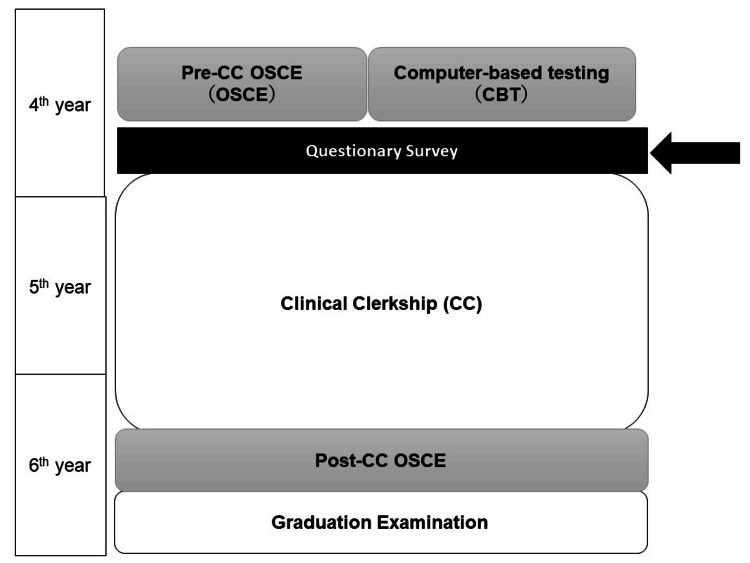
Curriculum course in our university and questionnaire timing on this survey Image Credit: Nobuyasu Komasawa

Japanese medical schools usually consist of a six-year study period. Students can enter medical school after graduating from high school and successfully passing an entrance exam. As with other medical schools in Japan, medical students at the Faculty of Medicine, Kagawa University, complete all basic and clinical medicine lectures and skill training before beginning a CC, typically in the fourth grade. In the sixth grade, students complete their advanced CC and take a graduation exam (Figure 1) [15].

OSCE content and evaluation

The OSCE, conducted before the CC, assesses various aspects of clinical competency. This OSCE comprises eight key themes: medical interviewing, head and neck examination, chest examination, abdominal examination, neurological examination, systemic and vital sign assessment, emergency response, and fundamental clinical techniques. The OSCE is organized into eight stations, encompassing a 10-minute medical interview at one station and physical examinations with basic skills at the remaining six stations, allocating five minutes for each 13. Examiners employ a checklist to evaluate communication, medical safety, and consultation skills accordingly. Every student undergoes examinations across all eight skill stations, and a total score is calculated based on the average performance across these stations.

CBT content and evaluation

The CBT consists of multiple-choice questions and extended matching items, and students are required to answer 320 questions about basic clinical knowledge over the course of six hours. The CBT consists of six themes: one basic competency required for doctors, two society and medicine, three medicine in general, four normal structures, function, condition, diagnosis, and treatment in each organ, five systemic physiological changes, condition, diagnosis, and treatment, and six basics of medical treatment (Table 1). The CBT includes clinical disciplines and related basic medicine knowledge. Scores for the CBT are machine-calculated, and the scoring rate was evaluated.

Statistical analysis

Statistical analyses were performed using JMP Pro version 13.2.1 software (SAS Institute Inc., Cary, NC, USA). The results were compared using the Mann-Whitney U test or Kruskal-Wallis test, followed by Scheffe's multiple comparisons. Data are presented as mean ± standard deviation. P-values <0.05 were considered statistically significant.

## Results

**Figure 2:**
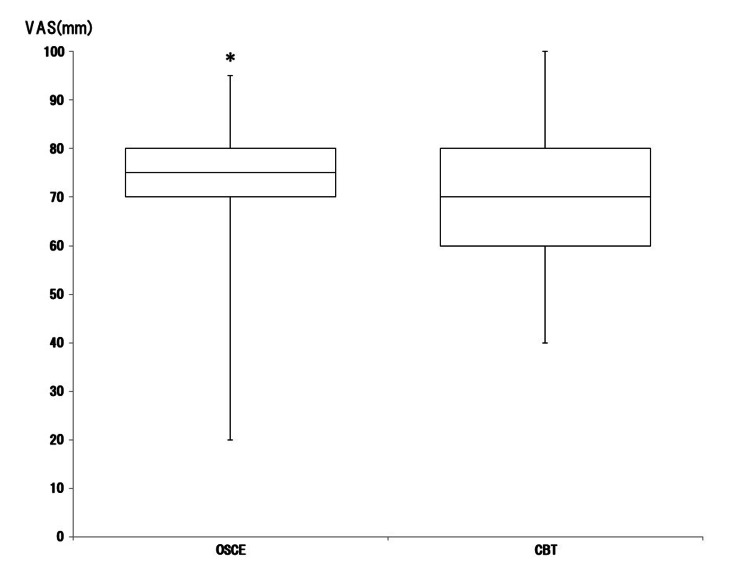
Box-and-whisker plot (median, interquartile range, and range) of subjective confidence in accomplishment on objective structured clinical examination (OSCE) and computer-based testing (CBT) in medical students using the visual analog scale (VAS), which ranged from 0 mm (extremely unconfident) to 100 mm (extremely confident) *p<0.05 was considered statistically significant Image Credit: Nobuyasu Komasawa

In total, 71 of 105 fourth-year students responded to the survey (response rate: 67.6%). Confidence in total OSCE and CBT in medical students is shown in Figure 2.

**Figure 3:**
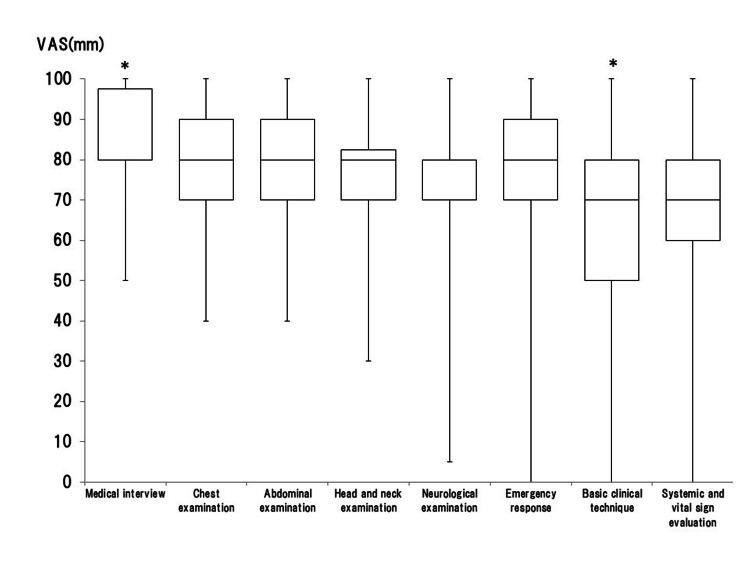
Box-and-whisker plot (median, interquartile range, and range) of subjective confidence in the eight themes of objective structured clinical examination (OSCE) in medical students using the visual analog scale (VAS), which ranged from 0 mm (extremely unconfident) to 100 mm (extremely confident) *p<0.05 was considered statistically significant between the groups Image Credit: Nobuyasu Komasawa

The confidence in accomplishment on OSCE was significantly higher than on CBT (p=0.014). Confidence in the eight themes of OSCE is shown in Figure 3.

**Figure 4:**
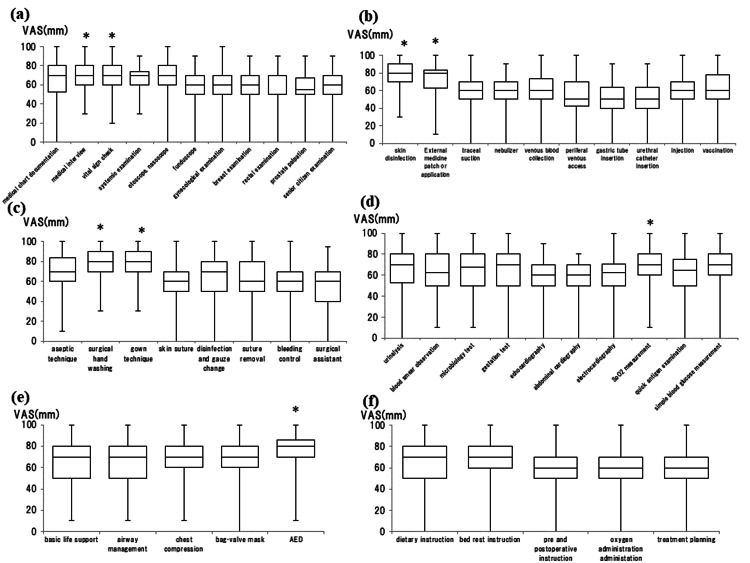
Box-and-whisker plot (median, interquartile range, and range) of subjective confidence in essential medical practices for clinical clerkship (CC) recommended by the "Report on Medical Practice During Clinical Clerkship" using the visual analog scale (VAS), which ranged from 0 mm (extremely unconfident) to 100 mm (extremely confident). (a) Medical examination, (b) general technique, (c) surgical technique, (d) laboratory examination, (e) emergency response, and (f) treatment *p<0.05 was considered statistically significant between the groups Image Credit: Nobuyasu Komasawa

The confidence in medical interviews was significantly higher than that of neurological examination (p=0.003) and basic clinical technique (p<0.001). The confidence in basic clinical technique was also significantly smaller compared to that in the chest or abdominal examination (p=0.014, p=0.010) and emergency response (p=0.025). Confidence in various skills in public CC is shown in Figure 4.

In medical examination, medical interview showed stronger confidence compared to gynecological, breast, or rectal examination and prostate palpitation (p<0.05, each). A vital sign check also showed stronger confidence compared to breast or rectal examination and prostate palpitation (p<0.05 each; Figure 4a). In the basic technique, skin disinfection showed stronger confidence compared to other techniques (p<0.05 each). External medicine application also showed stronger confidence compared to tracheal suction, nebulizer, peripheral venous access, gastric tube insertion, urethral catheter insertion, and injection (p<0.05 each; Figure 4b). On surgical technique, surgical hand washing and gown technique significantly showed stronger confidence compared to skin suture, suture removal, bleeding control, and surgical assistant (p<0.05 each; Figure 4c). The confidence in SpO2 measurement was significantly higher compared to chest or abdominal echocardiography (p=0.039, p=0.027; Figure 4d). As for emergency skills, confidence in AED was significantly higher compared to airway management (p=0.019; Figure 4e). There was no significant difference in treatment (Figure 4f).

**Figure 5:**
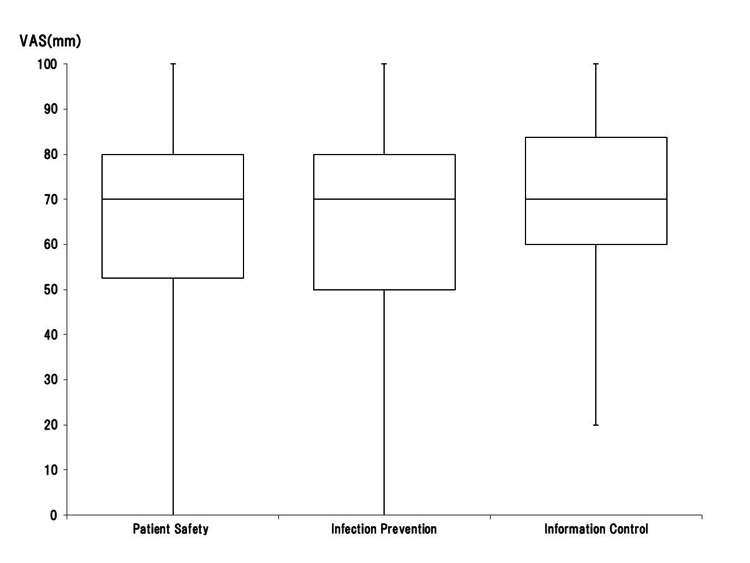
Box-and-whisker plot (median, interquartile range, and range) of subjective confidence in medical safety (patient safety, infection control, information management) using the visual analog scale (VAS), which ranged from 0 mm (extremely unconfident) to 100 mm (extremely confident) Image Credit: Nobuyasu Komasawa

Confidence in medical safety (patient safety, infection control, and information management) associated with CC is shown in Figure 5. While confidence in information　management　was high　compared　to　patient　safety　or　infection　control, there were no significant differences.

## Discussion

The objective of an integrated medical education system primarily centers around refining the educational experience for undergraduate medical students, enabling them to evolve into reliable healthcare professionals [16,17]. To attain this objective, the development of an efficient clinical training system that facilitates a seamless progression from undergraduate medical education to the acquisition of critical skills as student doctors is essential [11,18]. Both CBT and OSCE conducted prior to the CC are critical in ensuring that medical students possess the fundamental clinical skills necessary to engage in entrustable professional activities.

In our study, confidence in CBT, which plays a central role in knowledge, was smaller compared to that in OSCE. This tendency suggests that medical students have confidence in basic skills at the stage just before CC. In contrast, medical students showed less confidence in non-invasive medical practices compared to invasive or complex ones in various medical examinations, basic clinical techniques, or surgical technique regions. This result suggests that present skill training before CC is insufficient. As medical students are expected to perform entrustable medical practice as student doctors, systematic preparatory training not only during CC but also before CC is warranted.

A significant challenge in medical education within a clinical context is the time constraints faced by clinical educators, a result of national medical policies promoting a better work-life balance for doctors. It seems impossible for educators to provide sufficient clinical training for student doctors on essential medical practice. Overwork-related deaths have been a persistent issue in Japan for more than half a century, with frequent reports in the media about medical practitioners, especially those in training, succumbing to overwork. Doctors in Japan endure the longest working hours compared to professionals in other fields [19]. In response to this, Japan's Ministry of Health, Labor, and Welfare has recently made efforts to reform medical work schedules and initiated discussions on this matter. In Japan, medical professionals at academic medical institutions are encouraged not only to fulfill their routine clinical responsibilities but also to actively participate in research and educational endeavors [20]. Due to their strong work ethic, these professionals often accept long working hours, despite the adverse effects on their well-being. These roles are highly specialized and cannot be delegated to other healthcare staff, making it challenging to implement significant changes to their work schedules [20]. These circumstances contribute significantly to the complexities associated with achieving a streamlined and effective workstyle reform and systematic regulation of doctors' working hours in Japan. It's essential to consider not only medical regulations but also patient comfort and privacy when engaging in active clinical clerkship. Without effective measures, achieving a balance between work and personal life and maintaining the quality of CC becomes a challenge, conflicting with health policies.

One potential solution may lie in further embracing simulation-based education [21,22]. Student doctors can enhance their fundamental skills using simulation, potentially reducing the burden on supervising doctors in a clinical setting [23,24]. At present, medical students focus on basic skill training using simulation to pass OSCE before CC. Our result showed a tendency for medical students to show strong confidence in basic themes associated with OSCE but not in themes not related to it. As OSCE is a minimum guarantee for starting CC, introducing more simulation-based education on essential medical practice is warranted. Additionally, there are reports suggesting that simulation-based education could alleviate stress factors [25,26]. Therefore, intensive simulation-based training before and during clinical clerkship may serve as an effective solution, not only for enhancing medical safety but also for reducing the stress associated with clinical clerkship. As digital education technology such as on-demand classes evolves in the medical education field [27], it may be possible to expand the ratio of simulation-based education to clinical skill acquisition [28].

This study has several noteworthy limitations. Firstly, the data was obtained from a single institution, potentially limiting the generalizability of the findings to medical schools in other countries. Secondly, both male and female participants were included in the analysis. An exploration of gender differences concerning medical practice during clinical clerkship and the training environment could be an intriguing avenue for future research [29]. Thirdly, we solely conducted quantitative analysis on career design using the VAS and Likert scales to gauge awareness and attitudes toward career design [30,31]. In the future, it would be beneficial to complement these assessments with qualitative analyses such as interviews or text-mining analyses on portfolios [32].

## Conclusions

We examined the confidence of OSCE and CBT, which are prerequisites for CC for medical students who are just starting CC. We also evaluated the confidence in the medical practice and training environment during CC. While medical students showed stronger confidence in OSCE themes, they showed limited confidence in invasive medical practice during CC. These results suggest that medical students just before CC have less confidence in invasive medical practice during CC. As medical practice by student doctors becomes public, further systematic basic skill training is warranted.
